# A Prospective Natural History Study of Quitting or Reducing Gambling With or Without Treatment: Protocol

**DOI:** 10.2196/resprot.2727

**Published:** 2013-12-02

**Authors:** Vladyslav Kushnir, John A Cunningham, David C Hodgins

**Affiliations:** ^1^Center for Addiction and Mental HealthToronto, ONCanada; ^2^University of TorontoDepartment of Pharmaceutical SciencesToronto, ONCanada; ^3^Center for Mental Health ResearchAustralian National UniversityCanberraAustralia; ^4^University of CalgaryDepartment of PsychologyCalgary, ABCanada

**Keywords:** gambling, treatment, life events, motivational factors, natural recovery, prospective natural history study, longitudinal study

## Abstract

**Background:**

Only a small percentage of gamblers ever seek treatment, often due to stigma, embarrassment, or a desire to handle their problems on their own. While the majority of pathological gamblers who achieve remittance do so without accessing formal treatment, factors related to successful resolution have not been thoroughly explored.

**Objective:**

Employing a prospective natural history design, the study will therefore undertake an investigation to explore life events, motivating factors, and strategies used by problem gamblers to quit or reduce their gambling without formal treatment.

**Methods:**

Prospective participants (19 years or older) currently gambling at problematic levels with strong intentions toward quitting gambling will be directed to fill out a Web-based survey. Eligible participants will subsequently complete a survey that will assess: (1) types, frequency, and amount of money spent on gambling, (2) life events experienced in the past 12 months, (3) level of autonomous motivation for change, and (4) use of treatment services. Every 3 months for the duration of one year following the completion of their baseline survey, participants will be sent an email notification requesting them to complete a follow-up survey similar in content to the baseline survey. The four surveys will assess whether participants have experienced changes in their gambling behaviors along with positive or negative life events and motivations for change since the last survey. Individuals who are in the action and maintenance stages of quitting gambling at follow-up will be also asked about their techniques and strategies used to quit or reduce gambling. At 18 months post baseline, participants will be asked to complete a fifth and final follow-up survey that will also assess whether participants have experienced any barriers to change and whether they resolved their gambling to low risk levels.

**Results:**

The study has commenced in May 2013 and is currently in the recruitment stage. The study is scheduled to conclude in 2016.

**Conclusions:**

As this study will examine the active ingredients in natural recovery from gambling problems, the results will inform ways of promoting change among the large number of problem gamblers who do not seek treatment as well as improve treatment for those who do seek help. The information gained will also be useful in identifying effective self-help strategies for those who face challenges in accessing treatment, may be incorporated in standard treatment, provide brief intervention techniques, as well as inform relapse prevention strategies.

## Introduction

### Natural Recovery From Addictions

Natural recovery from addictions is not a recently recognized phenomenon. A variety of terms have been used to describe it including self-change, spontaneous remission, maturing out, and natural remission [1]. Recognition of natural recovery has been met with resistance because the majority of the research on addictive disorders has used clinical treatment samples and because the traditional disease model of addiction has typically regarded addiction as progressive and irreversible. However, over the past couple of decades, a significant amount of research has focused on exploring the natural course of various types of addictions. This research has revealed that recovery from addictions without formal treatment is common [2].

### Natural History Research of Problem Gambling

There are three main types of natural history research: (1) epidemiological studies that examine the prevalence of untreated change from an addictive behavior, (2) retrospective natural history research that recruits samples of former problem gamblers who quit or reduced their gambling at some point in the past and explores how they succeeded with this change, and (3) prospective natural history studies that recruit gamblers who intend to quit or reduce their gambling and follows them over time to explore factors associated with successful change. Each of these types of research has its strengths and weaknesses. Epidemiological survey research has the advantage of employing representative samples, but often lacks a “depth” of information that allows the researcher to explore factors associated with change. Slutske [3] used two epidemiological surveys from the United States and found that the majority of people who remitted from pathological gambling did so without accessing treatment or Gamblers Anonymous (only 7-12% sought treatment). Similar results have been noted in Ontario, Canada [4]. Retrospective research studies can recruit samples of people who had serious gambling problems that they dealt with in the past without treatment. Further, retrospective studies can go in depth into the factors that lead to the person’s recovery. In the gambling research area, this method has been employed in two studies [5,6]. The studies found that participants emphasized reasons for change such as emotional and financial consequences, hitting “rock bottom,” and issues related to self-image. In addition, both studies found that resolved problem gamblers with more severe gambling problems prior to resolution were more likely to have accessed treatment as compared to those with less severe problems prior to resolution. Finally, a study by Hodgins and el-Guebaly [5] found evidence for a life-events driven process of recovery without treatment such that resolved participants endorsed an increase in positive life events and a decrease in negative life events when comparing the time before to the time after resolving their gambling problems. The importance of this life-events driven process in recoveries without treatment has also been noted in retrospective research involving other addictive behaviors [7]. Specifically, in the context of alcohol-related problems, particular life events have been shown to contribute to recovery and sustained remissions to a much greater degree than maturing-out reasons or interventions from medical personnel or family members [8,9]. In this larger research area, motivation has been identified as the other main theme responsible for driving and maintaining change and recovery from ones’ addiction, particularly, internally driven recognition of the need to change [7,10]. Hodgins and el-Guebaly [5], in particular, have noted that in addition to life events, recovered gamblers attributed intrinsic/autonomous motivational factors such as using “will power,” establishing self-respect/goal commitment, and a sense of accomplishment/pride as those responsible for maintaining their state of change. While these cognitive/motivationally laden intrinsic factors may indeed contribute to maintaining a recovered state, past research was unable to address how intrinsic motivations help individuals recover naturally in the first place.

Retrospective natural history research, while being a powerful tool to investigate processes of change, suffers from the weakness of the potential of a recall bias (ie, the event under study often occurred many years in the past and participants’ recall may be faulty). While prospective natural history research cannot often explore resolutions in as much depth as retrospective studies, prospective research has the distinct advantage of circumventing difficulties with faulty recall of events because change from problem gambling occurs after the initial measure of the predictor variables. Further, the hypothesized factors believed to be important in predicting successful change (collected before the actual change was made) can be related prospectively to successful change in order to differentiate those people who actually succeed from those who relapse back to problem gambling.

Despite the strengths of a prospective research design for natural history research, very little research of this type has been conducted. In the area of alcohol research, prospective studies have been conducted by Tucker [11] and Cunningham [12] with Tucker examining the role of discretionary spending on alcohol as a predictor of success at resolution from alcohol problems, and Cunningham exploring the relative contributions of life events and motivation for change as predictors of successful long-term resolutions. For gambling, Hodgins and el-Guebaly [13] conducted the only relevant study in which relapse to pathological gambling was prospectively related to hypothesized precipitants. While the subjects employed in this study had primarily attended or were currently attending treatment (making the study less relevant to research on untreated recovery), the results are interesting in that the most frequently reported attributions prior to relapse had to do with cognitions about winning and feeling the need to make money. The present prospective natural history study will differ from that conducted by Hodgins and el-Guebaly, in that we will explore the factors related to successful resolution from gambling problems rather than those factors related to relapse to pathological gambling. In addition, we will recruit samples of treated and untreated participants in order to allow comparisons of these two pathways to change.

### Specific Aims

The prospective natural history study will attempt to explore factors that relate to successful recovery from gambling problems. By examining prospectively both treatment assisted and natural recovery participants in a community sample, this study will investigate and address the factors related to successful resolution and reduction of gambling behaviors. In addition, the study will also examine and identify techniques related to maintenance and successful recovery from gambling problems.

### Hypotheses

Following the present natural history literature on the topic of recovery from gambling problems, three primary hypotheses are made.

The first hypothesis, life events, states that participants who experience an increase in positive life events and a decrease in negative life events will be more likely to display reductions in their problem gambling severity.

The second hypothesis, motivational, states that participants who display autonomous motivation for change will be more likely to reduce their problem gambling severity as compared to those who display nonautonomous motivation for change.

The third hypothesis, severity, states that participants with more severe gambling problems will be less likely to succeed at their change without treatment as compared to those with less severe gambling problems.

## Methods

### Participants

Participants will be recruited using a comprehensive strategy employing newspaper, Web-based, and television advertisements. Prospective individuals, 19 years or older, will be directed to fill out a brief Web-based screener that assesses age, problem gambling severity, and attitudes and intentions toward quitting gambling according to the transtheoretical model (TTM) of behavioral change [14]. Eligibility of individuals will be determined by agreement to be followed-up, a current score of 5 or more on the Problem Gambling Severity Index (PGSI) [15], and seriously thinking of quitting or cutting back gambling within the next 6 months (contemplation stage) or 30 days (preparation stage). Participants who have ever used, are currently using, or are planning on using treatment for their gambling concerns will not be excluded from the study. These participants will instead be treated as a comparison group since the same natural history hypotheses are relevant to both treated and untreated problem gamblers.

### Study Design and Procedures

The prospective natural history study will recruit problem gamblers who are seriously thinking of quitting or cutting back gambling within the next 6 months or 30 days, and follow them over an 18-month period to examine factors and techniques related to quitting or reducing gambling with or without treatment. Potential participants, self-identified as seriously thinking of quitting gambling will be directed to log on to a website listed on the advertisement. Subsequently, individuals, will be directed to a webpage containing a consent form, where they will be asked to enter their email address and confirm that they have read and understood the research and their rights before proceeding to a brief Web-based screener. The standing research ethics board of the Center for Addiction and Mental Health has approved this study. The brief screener will assess age, problem gambling severity, and attitudes and intentions toward quitting gambling according to the TTM of behavioral change [14]. Individuals identified as 19 years or older, seriously thinking of quitting or cutting down their gambling in the next 6 months or 30 days, currently gambling at problem gambling levels (PGSI score of 5 or more will be used to determine problem gambling), and willing to be followed-up for the duration of 18 months will be deemed eligible for study participation [15]. In an effort to engage participants in the study and reduce the likelihood of loss at follow-up, participants who are identified as eligible, based on their responses to the Web-based screener, will be immediately notified that they will receive a paper consent form in the mail in a few days. These individuals will be sent a paper consent form to sign and return in a postage-prepaid envelope in order to be invited to complete the baseline survey. Participants deemed ineligible, as per the screener, will be told that only if they are found eligible will they be contacted to fill out the baseline survey.

**Figure 1:**
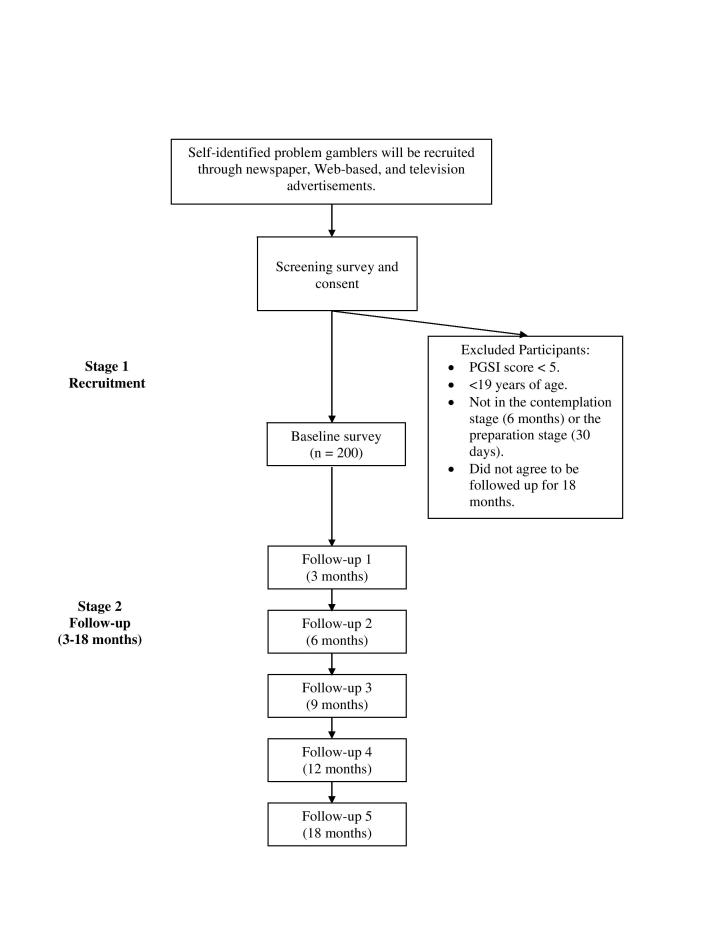
Overview of the study.

Following the return of a signed paper consent form, participants meeting eligibility criteria will be sent an email notification requesting them to fill out a baseline survey. The baseline survey will assess: (1) demographic characteristics and types, frequency, and amount of money spent on gambling, (2) life events experienced in the past 12 months (Life Events Questionnaire) [16], (3) level of autonomous motivation for change using the Treatment Self-Regulation Scale adapted for gambling to address intrinsic health change behavior [17-19]; guilt and shame proneness using the Test of Self-Conscious Affect, Version 3 (TOSCA-3) [20], (4) alcohol consumption using the Alcohol Use Disorder Identification Test-C (AUDIT-C) [21], (5) use of treatment services, and (6) past and current drug use and mental health diagnoses of Diagnostic and Statistical Manual of Mental Disorders-IV Axis-I disorders. Following the completion of the baseline survey, participants will be included as part of the study and will be sent an honorarium in the form of a $20 Amazon.ca gift certificate. At 3 months and every 3 months for the duration of one year following the completion of their baseline survey, participants will be sent an email notification requesting them to click on a hyperlinked Web address to complete a follow-up survey. Figure 1 shows the diagram of the study design. The four follow-up surveys will be similar in content to the baseline survey and will assess whether participants have experienced changes in their gambling behaviors along with positive or negative life events and motivations for change in the past three months. Individuals who are in the action and maintenance stages of quitting gambling at follow-up will be also asked of their techniques and strategies used to quit or reduce gambling using the Process of Change Questionnaire [22,23] modified for gambling. Following the completion of each follow-up survey, participants will be sent an additional $20 Amazon.ca gift certificate honrarium. In order to remain consistent and ensure that all participants are answering questions in the same manner, the point of reference for the four follow-up surveys will be life events, motivations, and gambling behavior in the last 3 months. At 6 months after completion of the fourth follow-up (18 months post baseline) survey, participants will be asked to complete a fifth follow-up survey. The fifth follow-up survey will be similar in content to other follow-up surveys, but it will also assess whether participants have experienced any barriers to change and whether they resolved their gambling to low risk levels. Following the completion of the fifth follow-up survey, participants will be sent an additional honorarium in the form of a $40 Amazon.ca gift certificate. In an effort to reduce loss to follow-up, at each follow-up period throughout the duration of the study, participants will be sent up to 3 automatic email reminders to complete their follow-up surveys.

### Description of the Measurement Tools Used

#### Screening Tools

The PGSI is a 9-item measure of problem gambling. Response choices for each of the PGSI questions are on a 4-point scale of “0 - never”, “1 - sometimes”, “2 - most of the time”, and “3 - almost always”. Totaled score cut-offs assign individuals to categories of “nonproblem gambler” (PGSI = 0), “low risk gambler” (PGSI = 1 to 2), “moderate-risk gambler” (PGSI = 3 to 7), or “problem-gambler” (PGSI >7). Most recently, Currie et al [24] have proposed a rescoring of the cut-offs for the low-risk (1-4) and moderate-risk (5-7) categories, showing better delineation between the two categories in a number of gambling-related dimensions. Our research will thus employ this revised PGSI scoring method, thereby decreasing chances of false positives and concurrently enabling us to test the reliability of the newly revised PGSI categories over the course of the study.

#### Content of the Baseline Survey

##### Types of Questionnaires

The baseline survey will assess the following: (1) types, frequency, and amount of money spent on gambling, (2) the life events experienced in the past 3 months as measured using a simplified version of the Life Events Questionnaire (LEQ) [16], (3) level of autonomous motivation for change will be measured using the Treatment Self-Regulation Scale (TSRQ) adapted for gambling to address intrinsic health change behavior [17,19,25,26], (4) use of treatment services, (5) demographic characteristics such as gross household income, (6) alcohol use using the 3-item AUDIT-C questionnaire [21], and (7) past and current drug use and mental health diagnoses.

##### The LEQ

The LEQ [16] assesses a total of 78 life events in eight categories-work, residence, marriage and intimate relationships, family and children, friendship and social activities, finances, physical health, and legal matters. The LEQ yields a frequency score for each category and for total positive and negative events. The authors (JC and DH) of this protocol have previously created a simplified version of the LEQ that can be self-administered and has had success in employing this scale in other prospective natural history research conducted by mail [8]. The modified questionnaire uses a total count of negative and positive life events, rather than the 8 summary scales relating to the different domains of life events. Pilot data has shown that these subscales were highly intercorrelated with the total life events summary scale (correlations ranged from 0.34 to 0.68; *P*<.001 in all cases), making the use of these subscales largely redundant with the total negative and positive life events subscales.

##### The TSRQ

The TSRQ is a scale based on the Self-Determination Theory [17,18], which assesses the degree of autonomous self-regulation regarding why people engage or would engage in healthy behavior. The questionnaire has been designed to be adapted to a range of different health behaviors and is readily modifiable to ask about motivation for change in gambling. The questionnaire presents participants with a question such as “The reason I would stop gambling permanently or continue not to gamble heavily is…” and asks them to rate preselected responses on a 7-point Likert scale of strongly disagree to strongly agree. Typically 15 items are used, assessing external motivation (4 items; eg, “Because others would be upset with me if I gambled”), introjected motivation (3 items; eg, “Because I would feel bad about myself if I gambled”), identified motivation (4 items; eg, “Because I personally believe it is the best thing for my health”), integrated motivation (2 items; eg, “Because it is consistent with my life goals”), and amotivation (3 items; eg, “I don’t really know why”). Autonomous or “internal” forms of motivation have been regarded as identified and integrated, whereas nonautonomous or controlled forms of motivation have been identified as external and introjected [17,19,25,26]. Amotivation on the other hand has been treated as a unitary concept that identifies a lack of an intent or a value in performing a given behavior [26]. Previous research using the TSRQ found that autonomous forms of motivation have been associated with behavioral outcomes such as active participation in an alcohol treatment program [27], long-term maintenance of weight-loss in a stringent program for patients who were initialy morbidly obese [28], change in tobacco use for adolescents [29], and long-term tobacco abstinence for adults [25], as well as adherence to medication regimens [30,31]. In contrast, nonautonomous motivation and amotivation have been linked to nonadherence to treatment and poorer health and well-being [19]. Reliability estimates for the autonomous and nonautonomous subscales have been shown to be excellent, with a mean Cronbachs alpha score of .91 and .82, respectively [32]. Other research further determined that across three different health-related behaviors (diet, exercise, smoking) the internal consistency for autonomous motivation subscales ranged from .85 and .93 and for nonautonomous motivation it ranged from .74 and .91 [19].

##### Shortened TOSCA-3

The shortened TOSCA-3 [20] will be used to measure participants’ propensity to experience shame and guilt at baseline. The test uses 11 brief scenarios depicting situations that would commonly elicit shame and/or guilt. Although the shortened version of the TOSCA-3 drops positive scenarios to eliminate the pride scales, the shame and guilt scales correlate .94 and .93, respectively with their corresponding full versions, thus supporting the utility of the abbreviated form [20].

##### The AUDIT-C

The AUDIT-C is a brief 3-item questionnaire that assesses alcohol misuse and tests for heavy drinking, active alcohol abuse, or alcohol dependence [21]. The response options for each item are scored 0-4 points, and possible AUDIT-C scores range 0-12 points. The AUDIT-C has been modified to a 3-item questionnaire from the original 10-item Alcohol Use Disorder Identification Test. The questionnaire exhibited high validity and reliability in many population samples, and has been validated in several countries by the World Health Organization [33].

#### Content of Follow-Up Surveys

##### The First Four Follow-Up Surveys

The follow-up surveys will assess identical constructs as in the baseline survey, including the PGSI as the primary outcome measure. The point of reference for the first 4 follow-up surveys will be events that occurred in the last three months. In addition, the Process of Change (PoC) Questionnaire [22,23] modified for gambling will be administered to individuals in the action and maintenance stages of quitting gambling at follow-up to assess techniques and strategies used to quit or reduce gambling.

##### The Fifth and Final Follow-Up

The fifth and final follow-up survey will assess identical constructs as the other follow-up surveys, however the point of reference will be events in the last six months. The survey will also assess obstacles to change using the Barriers to Change Questionnaire [34] with participants who have not quit or experienced a significant reduction in their gambling during the study. Further, success at resolving gambling problems by gambling at low risk levels will be determined by criteria identified by Currie et al [35].

##### The PoC Questionnaire

The PoC Questionnaire [22], originally designed to measure the change processes of smoking cessation, provides highly reliable measures of 10 processes of change, labeled: (1) consciousness raising, (2) dramatic relief, (3) self-liberation, (4) social liberation, (5) counterconditioning, (6) stimulus control, (7) self-reevaluation, (8) environmental reevaluation, (9) reinforcement management, and (10) helping relationship. The questionnaire has since been modified for use in other problem areas, with the gambling-modified version developed in 2001 to reflect factors and strategies used by recently resolved and active problem gamblers throughout the process of recovery [23]. It consists of 30 items querying how often the person has used a process in helping change gambling behavior, rated on a 5-point scale (1 = never, 2 = seldom, 3 = occasionally, 4 = frequently, 5 = repeatedly).

##### Barriers to Change Questionnaire

The Barriers to Change Questionnaire is a 28-item questionnaire previously developed by the authors (JC and DH) to assess barriers to change and delays to seeking treatment in problem gamblers [34].

##### The Low-Risk Threshold

In an effort to examine the relationship between gambling behaviors and gambling-related harm, Currie et al [35] conducted risk-curve analysis of the Canadian Community Health Survey - Mental Health and Well-being (Cycle 1.2) [36] to establish low-risk gambling limits. It was determined that the optimal low-risk threshold for gambling was gambling no more than three times per month, spending no more than $1000 CDN per year, and 1% of gross family income. This low-risk threshold did not change based on the definition of gambling-related harm; whether in terms of experiencing negative consequences or with a broader definition that included consequences and behavioral problems. The relationship between gambling activity and risk of harm has been further shown to be independent of gender, age, and socioeconomic status.

### Power and Sample Size

**Figure 2:**
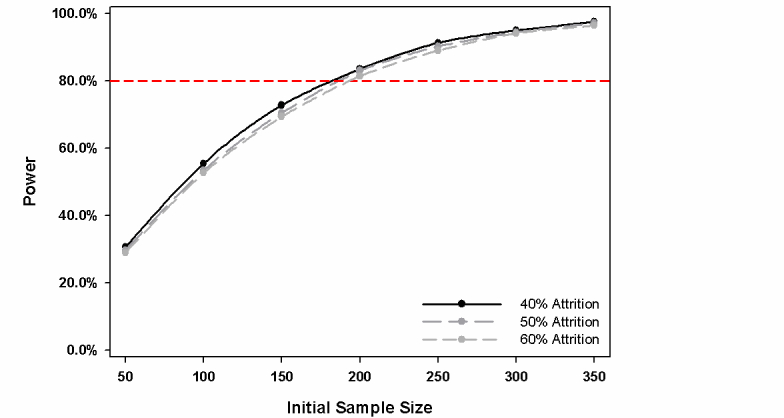
Initial sample size versus power.

To determine the number of study participants required to identify factors related to successfully quitting or reducing problem gambling behavior, a series of Monte Carlo simulations (with 10,000 replications per target sample size) were carried out with PGSI total scores used as the primary outcome measure. We assume that baseline means and standard deviations are similar to those reported by Bagby et al [37] (Sample 2). We further assume that repeated measures taken on the same individual over time would show a moderate degree of correlation (*r*=.25). The effects of our predictors of interest in this investigation will be assessed using mixed models. Assuming 40% attrition over the course of the study, we will have sufficient power (>80%) to detect a relationship between PGSI total scores and one or more of our predictors of interest with a sample of 200 study participants provided that the combined impact of these predictors on gambling behavior is associated with a coefficient of determination *ƒ* ^2^=0.02 or greater. Following the guidelines outlined by Cohen [38], this corresponds to a small effect size. Figure 2 shows that an initial sample of 200 study participants will provide us with sufficient power to detect a small effect even if attrition is higher than initially anticipated.

### Data Analysis

All analyses will be two-tailed and will be carried out at an alpha level of .05 using the SAS System for Windows v.9.3 (The SAS Institute, Cary, NC). Prior to analysis, the data will be screened to ensure that the underlying assumptions for all subsequent statistical procedures are met. Preliminary analyses will include *t* tests, analyses of variance, and correlations and will examine the relationship between PGSI total scores and all predictors of interest. Should any continuous predictors be associated with PGSI total scores in a nonlinear manner, we will investigate appropriate transformations, categorizations, and viable thresholds for piecewise analyses. Any demographic characteristics (age, gender, education, marital status, employment status, household income) found to be associated with gambling severity will be included as covariates in all subsequent analyses.

Our three primary hypotheses will be addressed using linear mixed models. Unlike traditional analyses that typically assume independence of observations, mixed models allow observations to be correlated and are appropriate for use in longitudinal analyses where repeated measurements are taken on the same study participants over time. Additionally, these models use all available data in an efficient manner (ie, partial information derived from subjects lost to follow-up may be included in the analysis and it is not necessary to restrict the analysis to study completers only). The ratio of positive to negative life events, measures of motivation, use of treatment services, severity of gambling problems, amount of money spent on gambling, gambling frequency, time, and any relevant demographic characteristics identified through our preliminary analyses will be included in these models as predictor variables. We will also investigate potential interactions between time, treatment status, and all remaining predictor variables. We will use a purposeful selection of covariates approach [39] to select the subset of main effects and interactions to be included in our final model. Exploratory analyses will further examine whether potential interactions between predictor variables are associated with gender differences, thereby possibly requiring stratification by sex.

## Results

The study has commenced in May 2013 and is currently in the recruitment stage. The study is scheduled to conclude in 2016.

## Discussion

As this study will examine life circumstances and motivational factors that play a role in successful resolution from gambling problems, the results will inform ways of promoting change among the large number of problem gamblers who do not seek treatment as well as improve treatment for those who do seek help. If altered life circumstances are closely associated with successful change from gambling problems (ie, the first hypothesis is supported), then this will imply that treatment should focus on providing the tools to help the person change their life circumstance (eg, develop social support, move to a new location, change leisure activities). If initial motivation for change is autonomous and a significant predictor of successful change (ie, the second hypothesis is supported), then this will tell us that treatment interventions could most profitably be focused on increasing such motivation for change. The information gained could be used to inform problem gamblers who are unlikely to seek treatment, and reinforce self-help techniques currently in place, by outlining potential targets or factors associated with successful recovery for problem gamblers. In addition, the information may be instrumental in informing relapse prevention strategies and could be incorporated as part of brief-intervention strategies or complimentary to standard treatment.

## Abbreviations

AUDIT-C: Alcohol Use Disorder Identification Test-C
LEQ: Life Events Questionnaire
PGSI: Problem Gambling Severity Index
PoC: Process of Change
TOSCA-3: Test of Self-Conscious Affect, Version 3
TSRQ: Treatment Self-Regulation Scale
TTM: transtheoretical model
